# Lying-In

**Published:** 1892-12-24

**Authors:** 


### LYING-IN.

**The City of London Lying-in Hospital**, City Road, E.C., has during the past year had more patients than in any previous year, and that with the most satisfactory results, not a single maternity death having taken place in the hospital since May, 1891; this fact is a sufficient proof of the care and attention bestowed upon the patients, and of the antiseptic treatment, which is fully carried out. The committee contemplate enlarging the hospital during the coming year by building additional dormitories, and a dining hall for the pupils and nurses. This will allow another ward for the use of the patients. To meet this outlay the committee confidently appeal to the public for additional donations and subscriptions. Mr. R. A. Owthwaite, secretary.

**Royal Maternity Charity**, 31, Finsbury Square, E.C.—One of the oldest and largest lying-in charities in Great Britain, attending some 4,000 poor women annually at their own homes, this charity appeals to all. Under the system of home attendance the limit to the charity's work is set only by the funds it has to deal with. Donations and legacies are greatly required. Secretary, Mr. T. W. Long.

